# Age and Gender Differences in Anthropometric Characteristics and Motor Performance of 3 through 6 Young Kids Aged (Pilot Study)

**DOI:** 10.3390/children10030590

**Published:** 2023-03-19

**Authors:** Almir Atiković, Ekrem Čolakhodžić, Edin Užičanin, Emilija Petković, Amra Nožinović Mujanović, Edin Mujanović, Jasmin Zahirović, Naida Mešković, Ana Lilić

**Affiliations:** 1 Faculty of Physical Education and Sport, University of Tuzla, 2. Oktobra 1., 75000 Tuzla, Bosnia and Herzegovina; 2 Faculty of Education, University Džemal Bijedić of Mostar, 88000 Mostar, Bosnia and Herzegovina; 3 Faculty of Sport and Physical Education, University of Niš, 18000 Niš, Serbia

**Keywords:** children, handgrip strength, dynamometer, hands tapping, legs tapping

## Abstract

Background: It is crucial to evaluate children’s motor coordination and strength to identify possible motor deficits on the right or left side of the body. However, whether a distinction exists in children aged 3–6 must be clarified. The goal of the current research was to investigate the differences in motor skills between preschool boys and girls, dominant and non-dominant hands or legs, in children of preschool age. (2) Methods: The present study was conducted on a sample of children (boys, n = 52; girls, n = 52; age range, 3–6 years). Three motor tests evaluated on both sides of the body served as the sample of factors used to measure athletic performance. Leg tapping (15 s), hand tapping (15 s), and a maximal hand grip strength (HGS) test kg. (3) Results: The study’s findings show no statistically significant variations in preschool boys’ and girls’ motor skills. Preschool girls had better results in the right leg tapping than preschool boys *t* (98) = 2.08; *p* ≤ 0.04. We found a significant difference between genders aged 3–4, 4–5, and 5–6 years. No correlation was found between the girls’ three variables and age. A small but significant positive correlation was found between dominant hand tapping and age *r*^2^ (52) = 0.21; *p* ≤ 0.01, dominant leg tapping and age *r*^2^ (52) = 0.20; *p* ≤ 0.01 and dominant HGS and age *r*^2^ (52) = 0.17; *p* ≤ 0.01. No noticeable differences were identified when comparing the dominant side with the non-dominant side in each group. The results show that most children prefer to use their right hand and right leg as their dominant sides. (4) Conclusion: The authors of this study focus on the functional (frequency of movements) and dynamic (differences in muscle strength between body sides) elements of asymmetry. Future studies should examine the influence of morphology on performance with the dominant or non-dominant body side.

## 1. Introduction

Children’s physical exercise levels have changed considerably in recent decades [1,2]. For example, children are increasingly being transported to school by vehicle or bus rather than cycling or walking [3], indoor pursuits are replacing outdoor active play, and involvement in organized sports is decreasing [4,5].

Hypokinesis, or the absence of physical activity, has been addressed from various health-related perspectives and documented in numerous studies worldwide. Hypokinesis is frequently associated with excess weight and obesity and can be found in preschool-aged children. Over the last two decades, many studies have been conducted on preschool children’s physical activity levels and motor abilities [6,7]. Generally speaking, boys and girls are regularly guided into different gender-based physical activities from an early age. It is important to note that motor abilities and habits develop the most in the period from the third to the tenth year of life, and the fact is that they can be mainly influenced in the preschool age, that is, from the fourth to the seventh year of life [8]. It is impossible to compensate for the lack of motor tasks or their total absence during a child’s growth in later stages of development, growth, and maturation. Specifically, as a child matures and grows, the impact of different kinesiological stimulations on them decreases gradually (the so-called critical phases). From ages 4–6 years, a child’s motor and intellectual development could be slowed by a lack of motor experience and chances for participation in kinesiological activities, all forms of planned physical exercise [9].

It is believed that hand and leg preference and asymmetries in motor coordination and speed emerge at the same time. In typically developing five- to seven-year-old youngsters, Denckla [10] discovered right-handed finger repetition was quicker than left-handed finger repetition. While the proportion of kids who performed quicker with their right hand rose with age, the size of this right-hand advantage shrank as people aged. Hand preference indicates that the preferred hand is faster and more coordinated than the nonpreferred hand. The favored hand starts to outperform the nonpreferred hand in speed and coordination throughout the first five years of life.

Hand grip strength (HGS) is a significant factor in growth, development, injury, exercise, rehabilitation, and regeneration [11]. Due to its importance as a marker for hand function, HGS has been assessed the most frequently. To establish baseline values to strive for when attempting to regain normal function and avoid early locomotor dysfunction in children, normative values of HGS must be established in a sample of healthy children [12]. Developing locomotor skills, the capacity to manage objects and nervous system maturity all improve in preschoolers. However, people’s basic motor abilities and physical fitness vary significantly [13,14]. Physical exercise is complex and involves several behavioral factors, including subjective elements (as in sports) and quantifiable elements (e.g., frequency, duration, and intensity). One of the most crucial movement skills is locomotion, which allows a child to manage an object in real-world circumstances, like throwing a ball. Therefore, a high level of movement proficiency could boost physical activity engagement [15].

There is a wealth of research on the connections between HGS and many essential medical indicators in various populations. Measurement of HGS is non-invasive, simple, and affordable. It may enable the investigation of acute changes in nutritional status and evaluation and prognosis of muscular strength in juvenile idiopathic arthritis, congenital myotonic dystrophy, and traumatic hand injuries [16,17,18,19]. Children’s height, weight, muscular mass, and bone density all impact HGS [20]. HGS is clinically essential for evaluating and comparing surgical techniques, tracking rehabilitation progress, documenting treatment reactions, and determining the degree of disability following injury. HGS is also used to evaluate the performance of athletes who rely on a proper level of grip strength to increase control and performance while minimizing potential injuries [21].

There is a wide range in HGS. To understand how grip strength changes with age, it is crucial to measure it during development. Without normative baseline data, we cannot distinguish between the impacts of growth, illness progression, and interventions, and surgical interventions considerably impact HGS [20]. Various dynamometers exist, including hydraulic, pneumatic, mechanical, and electronic devices [22]. These dynamometers’ mechanisms, performance, display mode, and energy supply differ. Among the most well-liked and extensively applied dynamometers is the TAKEI (Takei equipment) analog dynamometer grip A for infants (TKK5825 hand dynamometer). 

This study aimed to compare the motor skills of preschool boys and girls and establish how advanced their motor skills were. In addition, this study aimed to establish the dominant and non-dominant hand or leg in children of preschool age (3–6 years) and the percentage ratio.

## 2. Materials and Methods

### 2.1. Participants

This study included n = 104 preschoolers between the ages of 3 and 6 years (mean age of boys: 4.35 years, standard deviation: 0.88 years, and mean age of girls: 4.49 years, standard deviation: 0.73 years). The sample included 52 girls (50%) and 52 boys (50%). Children were enrolled in private preschools in Tuzla, Bosnia and Herzegovina. If a child had upper limb orthopedic or neuromuscular treatment, they were excluded, musculoskeletal problems, or upper extremity activities daily, neurological conditions that affected their upper extremities, or visual, auditory, or vestibular deficits.

### 2.2. Testing Procedures

#### 2.2.1. The Collected Data

Before providing written consent, kids’ parents were informed of the testing regulations and standards. To reduce inter-observer bias, all data were collected by the same examiner who trained all children to perform the procedures. Each child’s age and gender were recorded. Body weight and height were measured with a precision of 0.05 kg and 0.1 cm using the standard height scale and a typical digital weighing scale. The variables used to assess physical fitness [8,23] included three motor tests measured on each bodily side: frequency of movement (foot tapping, number/15 s, and hand tapping, number/15 s); and maximal strength (grip strength, kg). Hand tapping is tapping the fingers alternately against tapping boards for 15 s; the number of accurate cycles (one cycle is two taps) during the 15 s is tallied. Leg tapping is striking with the leg against tapping boards in a counterclockwise motion for 15 s. Again, correct cycles (one cycle is two taps) are recorded. Grip strength: squeeze a Takei dynamometer with a hand in a rotationally neutral position as hard as feasible; the grip’s width is independently adjustable; the experiment is done. Before the test, the examiner demonstrated standardized positioning for holding the hand dynamometer bulb. All participants were instructed: “squeeze the bulb as hard as you can for the count of three seconds.” A 2–5 s rest period was provided between trials, allowing the examiner to record the maximal HGS. Three trials were performed with each hand to avoid fatigue, alternating between dominant and non-dominant hands by TAKEI (Takei equipment) analog dynamometer grip A for infants (TKK5825).

#### 2.2.2. Data Analysis

We calculated the measures of central tendency (M and SD) and the multiple Pearson correlation coefficient (r2) to identify the variables correlated and then performed an independent *t*-test; the means on the left and right sides should be compared, girls and boys, and the dominant and non-dominant side of the body. Next, calculate the value of Cohen’s d and the effect size correlation, rYl, using the *t*-test value for a between-subjects *t*-test and the degrees of freedom. Results with a *p* ≤ 0.05 were considered significant. So each analysis was done using SPSS 23.0 for Windows (IBM Corporation, New York, NY, USA).

Cohen’s *d* = 2*t*/√(*df*) (1)

r_Yl_ = √(t^2^/(t^2^ + df)) (2)

### 2.3. Detection of Dominance

Each kid was instructed to sit at a proper table in front of the examiner while facing the chair. The youngster was told to take a pencil from the table and mark the white paper with a circular or a line. The examiner noted that the hand that drew the shape was the dominant hand [24]. Three-foot preference tasks were systematically given to each subject: kick the ball [25,26,27].

## 3. Results

The characteristics of the study group are described briefly in Table 1 according to the two groups. Of the n = 104 participants, there were 52 (50.0%) girls and 52 (50.0%) boys. The mean age difference between boys and girls was not statistically significant (t (102) = −89; *p* ≤ 0.373). However, these data did not show any significant gender differences. Table 2 presents the results of descriptive details relating to the motor skills of young boys and girls in preschool (ages 3–6). By observing the values of the *t*-test presented in (Table 1), it can be assumed that the *t*-test for independent samples was used to identify the differences between the two participant groups. The findings revealed significant differences between preschool boys and girls in one out of eight tested variables. Preschool girls had better results in the right leg tapping than boys (t (98) = −2.08; *p* ≤ 0.04; d = 0.42; r = 0.20). Across all seven tests; there were no noticeable differences.

**Figure 1:**
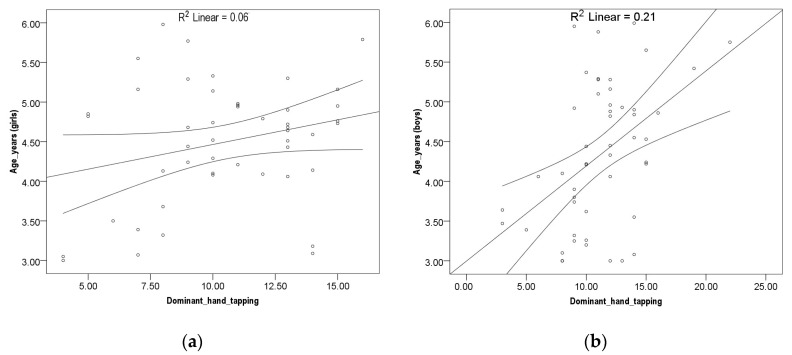
(**a**) Hand tapping girls. (**b**) Hand tapping boys.

**Figure 2:**
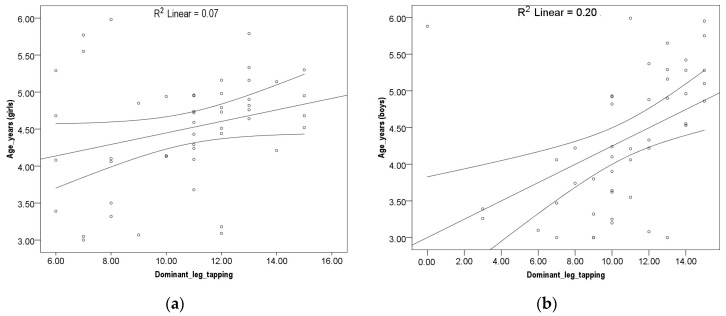
(**a**) Legs tapping girls. (**b**) Legs tapping boys.

**Figure 3:**
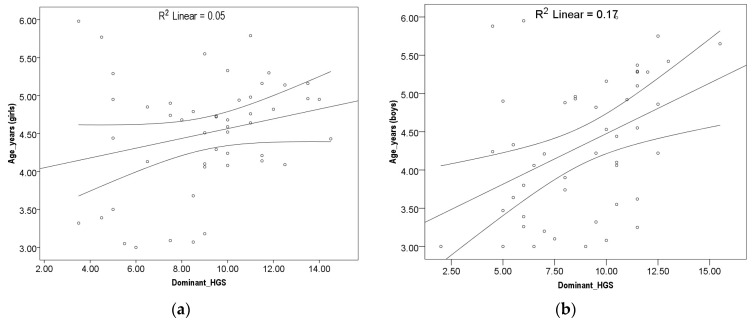
(**a**) Dominant HGS girls. (**b**) Non-dominant HGS boys.

No correlation was found between girls at all three variables and age. Results indicate no linear correlation. A Pearson correlation coefficient between boys’ age and dominant hand tapping, leg tapping, and hand grip strength tests was assessed with Pearson correlation coefficient, shown as a correlation in Figure 1, Figure 2 and Figure 3. A small but significant positive correlation was found between dominant hand tapping and age *r*^2^ (52) = 0.21; *p* ≤ 0.01), dominant leg tapping and age *r*^2^ (52) = 0.20; *p* ≤ 0.01 and dominant hand grip strength and age *r*^2^ (52) = 0.17; *p* ≤ 0.01.

In each of the groups examined, there were no noticeable differences between the dominant and non-dominant sides Table 2. During the measurement, it is obvious that the dominant side of the body is more on the right arm or leg than the left. The results show us that the dominant compared to the non-dominant looks like this: Hands dominant: boys’ right hand 85.1%, girls’ right hand 96.1%, legs dominant: boys’ right leg 80.9%, girls’ right leg 88.2%. In general, the left side of the body is used less already in early preschool age.

Age and motor skills are among the three age groups’ physical characteristics, which are presented in Table 3. values for hand tapping, leg tapping, and grip strength according to age.

As (Table 4), show statistical significant difference was found for girls in the: HTL-left hand 3–4/4–5 y. (*p* ≤ 0.015); LTR-right leg 3–4/4–5 y. (*p* ≤ 0.003); LTR-right leg 4–5/5–6 y. (*p* ≤ 0.033); LTR-left leg 3–4/4–5 y. (*p* ≤ 0.005); LTR-left leg 4–5/5–6 y. (*p* ≤ 0.007); HGSR-right hand 3–4/4–5 y. (*p* ≤ 0.002); HGSL-left hand 3–4/4–5 y. (*p* ≤ 0.000). *t*-test results also showed differences between boys and girls in the: HTR-right hand 3–4/4–5 y. (*p* ≤ 0.038); HTR-right hand 4–5/5–6 y. (*p* ≤ 0.040); HTL-left hand 3–4/4–5 y. (*p* ≤ 0.004); HTL-left hand 4–5/5–6 y. (*p* ≤ 0.046); LTR-left leg 3–4/4–5 y. (*p* ≤ 0.007); HGSR-right hand 3–4/4–5 y. (*p* ≤ 0.046); HGSR-left hand 3–4/4–5 y. (*p* ≤ 0.005).

## 4. Discussion

The hand-and-leg tapping HGS test is presently used because it is inexpensive and provides valuable information on muscle, nerve, bone, or joint disorders. However, it has also been linked to poor bone mineral density, poor cognition, and cardiovascular disease risk factors in kids and teenagers [28].

Boys were not significantly better at performing motor tests for predicting explosive and grip strength, while girls were not significantly superior in the frequency of simple movements. Our findings align with the findings provided by Bala et al. [6].

According to the study, all hand- and leg-tapping HGS scores significantly increased with age. Young children’s rapid increases in speed and grip power may be attributed to external variables like increased physical exercise with development or nutritional status, or they may result from variations in children’s growth rates concerning weight and height. Ploegmakers et al. [24] have confirmed these results. With advancing age, there is a linear trend toward better finger-tapping performance. The finger-tapping test for preschoolers did not significantly depend on the child’s gender. Exercise can help with speed and motor coordination [29]. One study included male and female pianists, and the authors found no significant effect of gender on finger-tapping speed [30]. This result might be explained by the training’s effect on balancing the differences between boys and girls. Performance, in terms of gender, among preschoolers likely depends on the nature of the task. For instance, studies on preschoolers’ visual-motor integration have consistently shown that females outperform boys [31].

It was discovered that the dominant hand’s grasp power was superior to that of the non-dominant hand. This might be brought on by the emergence of handedness between the ages of 3 and 6. As a result, the dominant hand is used for bodily tasks more often than the non-dominant hand. According to Souza et al. [32], the dominant hand’s grasp strength is 10% higher than the non-dominant hand’s grip strength in both genders and at all ages. This was in line with the current research findings, which verified that toddlers between the ages of 3 and 6 have stronger dominant hands.

Most research on how children acquire their motor abilities suggests that between the ages of 3 and 6 years, hand and leg preference begins to appear for several activities. By kindergarten, most typically developing kids regularly display a distinct hand preference, with about 90% doing most things with their right hand or leg. The remaining 10% of kids prefer using their left or right hands equally or exhibit delayed handedness. This suggests that age-related changes may persist throughout the school years [6,33,34]. Our research indicates identical results obtained through previous analyses. According to the findings, most children prefer to use their right hand and right leg as their dominant sides.

Participants produced more significant hand- and leg-tapping HGS values using their dominant hand than their non-dominant hand, regardless of age or gender. Such findings are consistent with recent findings from a cross-sectional survey of more than 2000 children and teenagers. Furthermore, these improved outcomes for the dominant hand over the non-dominant hand are unrelated to any particular hand form [35].

The maturity of the corpus callosum or the varying growth rates of the cerebral hemispheres may be markers for the development of motor skills, precisely the developmental pattern of asymmetries in left- vs. right-sided performance. Performance differences between activities on one’s favored side and those on their nonpreferred side seem to follow a developmental trajectory that starts tiny (infancy), then (from preschool through adolescence) becomes more and more dominant in favor of one’s developing preferred side [36], and lastly, after the age of nine, the ability to perform repeated finger tapping, groups of finger movements, and repetitive foot tapping decrease and even “adjusts” between both the right and left sides of the body [37].

In contrast to children with neurodevelopmental disorders, this growth pattern appears differently in healthy males and healthy girls [38]. This study investigated the relationship between different levels of physical fitness and cognitive functions in boys and girls. Step counts, physical education classes, and gender were all linked to particular brain results. These results may be crucial for supporting children’s school education and successful health promotion. Physical education classes for separate sexes may help kids’ cognitive development more. In order to confirm these findings, randomized studies are required [14]. Some authors [39] believe that for this age, improving motor skills and other working conditions (like water) can have better effects on developing motor skills. The authors conclude that a water initiation program integrating educational methodologies based on motor games is more effective than a traditional program based on motor repetitions.

## 5. Conclusions

In this article, the authors highlight the dynamical (differences in muscle strength between body sides) and functional (frequency of movements) aspects of asymmetry. Future studies should examine the influence of morphology on performance with the dominant or non-dominant body side. Early childhood education curriculum planners should be conscious of this and include fine motor training and activities on both the left and right sides of the body as a crucial component of the curriculum. The findings and data from this study have the potential to improve physical education programs that have a direct influence on children’s social and psychological health. This is due to the importance of hand holding in children’s play, handwriting, daily life, and sports activities.

## Figures and Tables

**Table 1:** *t*-test gender differences in anthropometric characteristics and motor performance of 3- to 6-year-old children.

Variable	G	n	M	SD	Skew	Kurt	K-S	F	*p*	t	df	*p*	d	r
BW	B	52	106.93	7.66	−0.70	0.86	0.94	0.30	0.58	−0.92	101	0.35	ns	ns
	G	51	108.36	8.16	0.07	−0.29	0.20			−0.92	100.31	0.35		
BH	B	52	17.98	2.45	−0.06	0.27	0.20	5.01	0.02	−1.37	101	0.17	ns	ns
	G	51	18.86	3.87	1.23	2.43	0.05			−1.37	84.37	0.17		
HTR-right hand	B	52	11.17	3.59	0.49	0.35	0.17	3.29	0.07	0.72	102	0.46	ns	ns
	G	52	10.71	2.82	−0.51	0.26	0.20			0.72	96.63	0.46		
HTL-left hand	B	52	10.32	3.55	−0.23	−0.15	0.54	5.03	0.02	−0.28	102	0.77	ns	ns
	G	52	10.50	2.50	−0.25	−0.20	0.05			−0.28	91.51	0.77		
LTR-right leg	B	49	10.22	3.03	−0.58	0.21	0.59	1.28	0.26	−2.08	98	0.04	0.42	0.20
	G	51	11.37	2.46	−0.48	−0.34	0.20			−2.07	92.45	0.04		
LTL-left leg	B	49	9.48	3.25	−0.58	0.36	0.82	5.78	0.01	−1.58	98	0.11	ns	ns
	G	51	10.39	2.40	0.13	−0.39	0.20			−1.57	88.17	0.11		
HGSR-right hand	B	51	8.89	3.34	−0.25	−0.61	0.20	2.98	0.08	−0.11	101	0.91	ns	ns
	G	52	8.95	2.58	−0.12	−0.95	0.07			−0.11	94.13	0.91		
HGSL-left hand	B	51	8.89	2.79	−0.36	−0.63	0.05	0.71	0.39	0.39	101	0.69	ns	ns
	G	52	8.69	2.45	−0.13	−0.75	0.20			0.39	98.84	0.69		

Abbreviation: G, gender; B, boys; G, girls; ns, not significant.

**Table 2:** Comparison of a body’s dominant (D) and non-dominant (ND) sides in boys and girls of 3- to 6-year old children.

Variable	D/ND	n	M	SD	Skew	Kurt	K-S	F	*p*	t	df	*p*	d	r
Body weight (kg)	D	88	18.48	3.32	1.15	3.13	0.05	0.42	0.51	1.05	95	0.29	ns	ns
	ND	9	17.27	2.35	−0.01	−1.142	0.20			1.39	11.16	0.18		
Body height (cm)	D	88	107.92	7.99	−0.29	0.34	0.20	0.00	0.95	0.02	95	0.35	ns	ns
	ND	9	105.34	7.44	0.34	−1.15	0.20			0.98	9.98	0.34		
Hand tapping 15 s (freq.)	D	89	11.04	3.26	0.18	0.74	0.05	0.58	0.44	1.76	96	0.08	ns	ns
	ND	9	9.00	3.74	−0.43	−1.53	0.20			1.58	9.27	0.14		
Leg tapping 15 s (freq.)	D	81	10.77	2.77	−0.48	0.09	0.06	3.49	0.06	0.84	94	0.39	ns	ns
	ND	15	10.06	3.97	1.37	1.59	0.07			0.66	16.62	0.51		
Hand gripstrength (kg)	D	86	10.84	3.01	−0.37	−0.43	0.05	1.39	0.24	0.01	95	0.98	ns	ns
	ND	9	8.88	2.20	−0.01	1.19	0.20			0.01	9.20	0.98		

Abbreviation: ns—not significant.

**Table 3:** Descriptive characteristics of the participants.

Variable	Groups	Girls						Boys					
	Age	*n*	M	SD	Skew	Kurt	K-S	*n*	M	SD	Skew	Kurt	K-S
BW	3–4 y	8	14.70	1.24	−1.87	4.20	0.06	13	100.11	6.74	−1.60	2.73	0.20
	4–5 y	34	18.67	2.62	0.96	1.75	0.06	27	108.051	5.99	0.15	−0.19	0.20
	5–6 y	10	22.82	4.97	0.59	0.88	0.20	12	111.79	7.37	−0.31	−0.61	0.20
BH	3–4 y	8	98.31	3.55	0.48	1.37	0.17	13	16.34	2.55	−1.10	2.71	0.20
	4–5 y	34	108.73	6.49	−0.46	0.72	0.20	27	18.08	1.90	−0.58	−0.08	0.05
	5–6 y	10	115.19	8.29	−0.20	−0.59	0.20	12	19.54	2.54	−0.98	1.28	0.20
HTR-right hand	3–4 y	8	9.00	1.51	0.99	1.63	0.15	13	9.00	2.54	−0.21	−0.61	0.20
	4–5 y	34	10.79	2.98	−0.85	0.67	0.15	27	11.11	3.05	−0.20	−0.79	0.07
	5–6 y	10	11.80	2.61	−0.38	0.05	0.20	12	13.66	4.27	0.31	0.04	0.07
HTL-left hand	3–4 y	8	8.50	1.19	0.00	−1.45	0.20	13	7.61	2.69	0.72	0.83	0.20
	4–5 y	34	10.64	2.29	−0.80	0.88	0.05	27	10.51	2.84	−0.71	0.91	0.20
	5–6 y	10	11.60	3.16	−0.69	−0.25	0.05	12	12.83	3.99	−1.08	1.14	0.05
LTR-right leg	3–4 y	8	8.87	1.55	0.27	−1.00	0.20	12	7.91	2.35	−0.28	1.01	0.20
	4–5 y	33	11.42	2.16	−0.86	1.19	0.10	26	10.38	2.43	−0.88	2.46	0.05
	5–6 y	10	13.20	2.44	−2.06	5.02	0.06	11	12.36	3.44	2.28	−6.07	0.05
LTL-left leg	3–4 y	8	8.00	1.51	−0.33	−1.48	0.16	12	7.16	1.69	0.22	0.45	0.18
	4–5 y	33	10.33	2.05	0.11	0.55	0.11	26	9.73	2.89	−0.54	0.65	0.12
	5–6 y	10	12.50	2.27	−0.85	0.09	0.20	11	11.45	3.98	−2.76	8.51	0.05
HGSR-right hand	3–4 y	8	6.31	1.25	0.277	0.288	0.20	12	7.12	3.28	0.10	−0.72	0.20
	4–5 y	34	9.26	2.37	−0.08	−0.35	0.20	27	9.18	2.68	−0.45	−0.48	0.19
	5–6 y	10	10.03	2.87	−1.52	0.91	0.05	12	10.00	4.24	−0.60	−0.56	0.20
HGSL-left hand	3–4 y	8	5.75	1.87	1.00	1.69	0.20	12	6.70	2.58	0.10	−1.47	0.16
	4–5 y	34	8.98	2.19	0.07	−0.65	0.20	27	9.17	2.33	−0.69	0.01	0.13
	5–6 y	10	10.05	1.96	−0.83	1.08	0.20	12	10.45	2.81	−0.73	0.16	0.20

**Table 4:** Age differences in static and dynamic tests of preschool children.

Variable		Girls							Boys						
		Levene’s Test		*t*-Test					Levene’s Test		*t*-Test				
		F	*p*	t	df	*p*	d	r	F	*p*	t	df	*p*	d	r
HTR	3–4/4–5 y.	3.48	0.06	−1.64	40	0.109	ns	ns	1.29	0.26	−2.15	38	0.038	−0.69	0.32
	4–5/5–6 y.	0.14	0.70	−0.96	42	0.342	ns	ns	1.08	0.30	−2.12	37	0.040	−0.69	0.32
HTL	3–4/4–5 y.	1.33	0.25	−2.54	40	0.015	−0.80	0.37	0.07	0.78	−3.07	38	0.004	−0.99	0.44
	4–5/5–6 y.	2.02	0.16	−1.05	42	0.297	ns	ns	0.92	0.34	−2.06	37	0.046	−0.67	0.32
LTR	3–4/4–5 y.	0.68	0.41	−3.12	39	0.003	−0.99	0.44	0.01	0.89	−2.93	36	0.006	ns	ns
	4–5/5–6 y.	0.00	0.99	−2.20	41	0.033	−0.68	0.32	0.65	0.42	−1.99	35	0.054	ns	ns
LTL	3–4/4–5 y.	0.18	0.66	−3.00	39	0.005	−0.96	0.43	3.47	0.07	−2.84	36	0.007	−0.94	0.42
	4–5/5–6 y.	0.42	0.51	−2.85	41	0.007	−0.89	0.40	0.00	0.99	−1.47	35	0.148	ns	ns
HGSR	3–4/4–5 y.	3.14	0.08	−3.38	40	0.002	−1.06	0.47	1.01	0.32	−2.06	37	0.046	−0.67	0.32
	4–5/5–6 y.	0.21	0.64	−0.85	42	0.397	ns	ns	4.06	0.05	−0.72	37	0.472	ns	ns
HGSL	3–4/4–5 y.	1.10	0.30	−3.84	40	0.000	−1.21	0.51	0.57	0.45	−2.95	37	0.005	−0.96	0.43
	4–5/5–6 y.	0.72	0.40	−1.37	42	0.175	ns	ns	0.13	0.71	−1.48	37	0.147	ns	ns

Abbreviation: ns—not significant.

## Data Availability

Other authors can get more materials and the SPSS version data from the authors.
